# Chronic Exertional Compartment Syndrome in a Fire Captain

**DOI:** 10.7759/cureus.27321

**Published:** 2022-07-27

**Authors:** Radhika Thakkar, Sydney Tran, Monica Gillie, Jeffrey Anderson

**Affiliations:** 1 Orthopedic Surgery, St. George’s University, School of Medicine, St. George, GRD; 2 Family Medicine, Stanford Health Care, San Jose, USA; 3 Orthopedic Surgery, O'Connor Hospital, San Jose, USA

**Keywords:** fasciotomy, lower extremity, chronic exertional compartment syndrome, compartment syndrome, orthopedic surgery, orthopedics

## Abstract

Chronic exertional compartment syndrome (CECS) is a commonly missed diagnosis. It is caused by an increase in intramuscular pressure which subsequently impedes local tissue perfusion and function. It disproportionately occurs in young females; however, the diagnosis should not be excluded in other demographics. We present a case of CECS in an otherwise healthy 53-year-old male fire captain. He presented with pain upon exertion and neurological deficits in the anterior compartment of his bilateral legs that impacted his occupation and daily functioning. Following fasciotomy, the patient returned to work with complete resolution of pain and neurological deficits. This review seeks to describe the prevalence, etiology, diagnostic criteria, differential diagnosis, and management of CECS of the lower extremities, as described in the literature.

## Introduction

Compartment syndrome is an increase in intramuscular pressure which subsequently impedes local tissue perfusion and function. Compartment syndrome can be further classified as acute and chronic. Acute compartment syndrome is a medical emergency and is commonly caused by traumatic injury to the extremity. This case focused on chronic exertional compartment syndrome (CECS), which is a reversible form of compartment syndrome that is also due to increased pressure within a muscle compartment caused by strenuous physical activity. In contrast to acute compartment syndrome, CECS is non-emergent, not necessarily surgically managed, and classically occurs in young, endurance athletes, especially runners [1].

The most common theory regarding the pathophysiology of CECS involves an elevation of interstitial pressure within the closed fascial compartment of the extremities [2]. During exercise, the muscle volume expands while the osseofascial compartment does not expand. The volume of muscles can expand by as much as 20% during exercise [3,4]. In the scenario of CECS, the fascia cannot accommodate this change in volume, thereby decreasing perfusion and oxygenation to the muscles during exercise. Decreased perfusion causes ischemia-related pain and neurological deficits in the affected compartment [5].

CECS can occur in the forearms, thighs, hands, and feet, but these presentations are seen in only 5% of cases. Moreover, 95% of cases occur in the legs, most often in the anterior or lateral compartments, followed by the deep posterior and superficial posterior compartments [6-9]. The anterior compartment contains the anterior tibial artery and vein and deep peroneal nerve. The lateral compartment contains branches of the anterior tibial artery and vein and the superficial peroneal nerve. The location of symptoms has been shown to align with the affected compartment’s nerve distribution.

Most literature supports that CECS is most often seen in women with a mean age of 24, and over 90% of cases present in athletes [10]. Other predisposing factors may include previous trauma causing muscle contusion or fracture leading to callus formation or muscle hematoma, all of which are space-occupying at the level of the muscle [11,12]. Individuals most commonly experience bilateral shin pain or calf pain, described as dull, aching, squeezing, or cramping, which occurs during exercise and is relieved during rest [2]. Complete resolution of symptoms occurs 10 to 20 minutes after the activity is stopped and is characteristically reproducible. CECS may also cause paresthesias, numbness, and foot drop due to compression of the nerves in the affected compartments. Patients also often have bulky, well-developed calves and associated muscle hernias or other fascial defects [13-15].

CECS can be ruled in by the clinical picture of reproducible pain with exertion among other clinical signs, but the gold standard for diagnosing CECS involves measuring intracompartmental pressures with a handheld manometer. Those with CECS often have normal compartment pressures at rest, but elevated compartment pressures during activity; therefore, pressures can be measured at rest prior to activity and/or at one and five minutes post-activity. One of the three following criteria must be met to fulfill the diagnosis of CECS: (1) resting pressure >15 mmHg, (2) one-minute post-activity pressure >30 mmHg, and/or (3) five-minute post-activity pressure >20mmHg.

After the diagnosis of CECS, there are both surgical and non-surgical management options. Non-surgical interventions include using shoe inserts, reducing training volume or workload, icing the area after exertion, and gait retraining [16,17]. For patients who are experiencing symptoms despite conservative treatment, surgical intervention is the next best option. This involves fasciotomy of the affected compartment.

## Case presentation

We present the case of a 53-year-old male with a complaint of persistent calf pain for the past several months. The patient had a non-contributory medical history of obstructive sleep apnea and diverticular disease. His surgical history was related to his diverticular disease and a biceps tendon repair. The patient was a fire captain, and his job required repetitive climbing and exercising. He stated that his pain would begin within five minutes of climbing stairs, running, and even walking long distances, sometimes leading to numbness in his feet and foot drop. Symptoms were unrelieved with over-the-counter anti-inflammatory medications. He was a non-smoker and drank minimal alcohol. He denied any recent history of trauma, significant lower extremity injuries, or back pain. He also denied a history of prior deep vein thromboses, cancer, coagulopathies, or pale or discolored feet and/or toes. The patient also stated that he had never experienced similar symptoms in the past.

On initial examination, the patient had a normal neurologic examination, but upon palpation, he had focal tenderness and firm, tight compartments in bilateral anterior legs. There was no radiation of pain and no associated joint pain. No erythema, diminished pulses, or asymmetric calf circumferences were noted. The patient had a body mass index of 36.77 kg/m^2^ and had bulky, well-developed calves at the time of examination.

A presumptive diagnosis of CECS was made based on clinical presentation alone. We opted to confirm our diagnosis by measuring compartment pressures. The patient was instructed to reduce his physical activity during work until his compartment pressures were measured. The patient was brought into the operating room to undergo measurements of his bilateral lower leg compartments. We had the patient exercise until his pain and numbness began, and then using a sterile technique, we used a local anesthetic and inserted a handheld compartment measuring device into the affected compartment in each leg. Compartment pressures measured 51 mmHg on the left leg and 47 mmHg on the right leg, supporting one of the three criteria for CECS and confirming the diagnosis.

After the measurements were collected, the patient returned for a preoperative visit to review the results. He reported worsening calf pain and that his symptoms were beginning to affect his daily life outside of work. The patient was brought in for a bilateral fasciotomy three weeks after measurements were conducted. Selective release of the anterior compartments was performed under general anesthesia and tourniquet. Two-inch longitudinal incisions were made over the left and right anterior compartment, and the fascia was released by passing scissors between the fascia and the muscle down to the ankle and up to the knee. Careful dissection was performed to ensure that proper fascial release and decompression had been conducted. The fascia was left open on both legs, and subcutaneous tissue and skin were closed. A compressive dressing was applied. The patient tolerated the procedure well and was asked to follow up in two weeks.

At the two-week follow-up, the right leg was doing well, but the left leg was mildly swollen and the wound was pushed open slightly. He was advised to wash the wound with soap and water twice a day and to keep Band-Aids over it. At the four-week follow-up, the patient reported that he was able to go up and down the stairs again and felt like his life was back to normal. Wounds were well-healed and he was neurovascularly intact with no tenderness.

## Discussion

CECS is characterized by exercise-induced, reproducible localized pain and even nerve deficits in severe cases. Although CECS is most often seen in the legs, it can be seen in the upper extremities and is generally seen in younger, female athletes. However, CECS is a commonly missed diagnosis, especially if the patient’s demographic is not typical for this diagnosis.

When a patient describes leg pain, differential diagnoses are determined by the location of the pain and may involve musculoskeletal, vascular, or neurologic systems. Malignancies and infectious etiologies are also possible. One of the most common causes of leg pain is tibial periostitis, also known as shin splints, which is also triggered by physical activity and relieved with rest. However, the pain usually begins one to three days after the trigger and leg compartments are not tense. One may also suspect a tibia or fibula stress fracture; however, pain from a stress fracture does not occur immediately after exertion and may be seen on advanced imaging studies. Radiculopathies can be ruled out by clinical presentation as they generally cause pain that radiates from the source of compression at rest and during activity, most commonly from the lumbar spine and into the thigh and leg. There may also be burning sensations and atrophy of muscles innervated by the affected nerve. Vascular pathologies, such as deep vein thrombosis (DVT), can also be ruled out by history and clinical presentation. DVT generally presents with unilateral swelling in the posterior calf with erythema or discoloration and warmth. Other differential diagnoses may include fascial herniation, popliteal nerve entrapment, tendinopathies, vascular claudication, or a bone tumor. In addition, bilateral versus unilateral presentation can help exclude several differentials.

## Conclusions

Patients and physicians simply may not be aware of the prevalence of CECS, therefore, missing opportunities for pain relief and prevention of symptoms. Patients who remain untreated may develop significant tissue ischemia, irreversible swelling, and escalating pain. The gold standard for making a diagnosis involves measuring compartment pressures. There are ongoing studies for non-invasive techniques; however, the current method has high specificity and an increased chance of positive outcomes. Surgical release is the standard of care for patients with moderate-to-severe CECS and for patients whose symptoms are refractory even after eliminating the trigger. Open fasciotomy of the anterior compartment is successful in a majority of patients, as seen in our patient, which leads us to conclude that surgical management is a reliable method for the relief of CECS symptoms. We hope to remind clinicians that CECS may present at an atypical age and gender, and we should be proactive with promoting surgical intervention, as it can significantly improve patient quality of life.
